# SS31 Ameliorates age-related activation of NF-κB signaling in senile mice model, SAMP8

**DOI:** 10.18632/oncotarget.14077

**Published:** 2016-12-21

**Authors:** Zhi-Hua Hao, Yue Huang, Mei-Rong Wang, Tian-Tian Huo, Qian Jia, Rong-Fang Feng, Ping Fan, Jian-Hua Wang

**Affiliations:** ^1^ Department of Neurology, Hebei General Hospital, Shijiazhuang, Hebei, China; ^2^ Graduate School,Hebei Medical University, Shijiazhuang, Hebei, China; ^3^ School of Medical Sciences, Faculty of Medicine, UNSW Australia, Sydney, Australia

**Keywords:** aging, SAMP8, inflammation, SS31, NF-κB, Gerotarget

## Abstract

Aging has been attributed to oxidative stress and inflammatory response, in which NF-κB and Nrf2-ARE signaling pathways play significant roles. Senescence accelerated mouse prone 8 (SAMP8) is generally used an animal model for aging studies. Here, we investigated the NF-κB and Nrf2-ARE signaling pathways in SAMP8 brains at different ages and their responses to SS31 peptide treatment. Thirty six SAMP8 mice were separated into aging groups and SS31-treatment groups. The hippocampus from each mouse was dissected for RNA and protein extraction. Cytokines and ROS levels were measured using ELISA and standardised method. Gene expressions of NF-κB, Nrf2 and HO-1 were measured by RT-qPCR. Total protein amount of NF-κB and HO-1, as well as the concentrations of nuclear and cytoplasmic Nrf2 were measured using Western blots. Our data showed that aging could activate both NF-κB and Nrf2-ARE signaling pathways, which could be suppressed and activated by SS31 treatment respectively. Regression analysis revealed that NF-κB gene expression was the most important parameter predicting aging process and SS31 treatment effects in SAMP8. Our findings suggested that SS31 treatment may modulate the inflammatory and oxidative stress status of the aged brains and exert protective effects during brain aging.

## INTRODUCTION

Aging, associated with cognitive decline, increases the risk of neurodegenerative disorders and leads to irreversible alterations in humans as well as in experimental animals [1, 2]. In this process, brain exhibits a progressively increased inflammatory status, which is called as “inflamm-aging” [3]. Chronic neuroinflammation and oxidation are neurotoxic and contribute to the pathogenesis of several aging-related diseases such as Alzheimer's disease (AD), Parkinson's disease, etc. [4].

The eukaryotic transcription factor nuclear factor-kappa B (NF-κB) is a master regulator in response to many pathological conditions, such as infectious agents and cellular stress [5]. NF-κB family is composed of homo- and heterodimeric complexes of Rel protein family numbers, consisting of NF-κB1(p105), NF-κB2 (p100), Rel B, c-Rel and Rel A (p65), which are regulated by IκB family members [6]. Among them, p65/p50 heterodimer is the best-studied and most abundant. Under normal conditions, p65/p50is maintained in the cytoplasm by inhibitor molecule IκB. Once activated by a variety of extracellular stimuli, p65/p50 complex is liberated and moved into nucleus. In nucleus, p65 drives expression of its target genes, with pro-inflammatory cytokines, growth factors, chemokines and adhesion moleculesencoded to regulate cell division and apoptosis [7-9]. The activation of NF-κB might play a major role in aging organisms [10]. While NF-κB functions as a cellular survival mechanism in acute responses, this transcription factor may cause neuro-inflammation and contribute deleteriously to age-related disorders [10].

Nuclear factor erythroid 2-related factor 2 (Nrf2) is another transcription factor essential for the regulation of genes to guard against oxidative stress [11]. In normal conditions, Nrf2 is sequestered in the cytoplasm and associated with the actin anchored Kelch-like ECH-associated protein 1 (Keap 1) [12]. Once activated, Nrf2 disassociates from Keap1 and translocated into nucleus from the cytoplasm to activate ARE-dependent transcription factors, up-regulating a battery of cytoprotective genes mainly phase IIenzymes such as heme oxygenase-1 (HO-1) [13, 14].

Senescence accelerated mouse prone 8 (SAMP8) is a strain of mice showing accelerated aging, which exhibits many behavioral characteristics of aging, such as cognitive decline, slow movement and loss of interest to the environment [15]. Senescence-accelerated mouse-resistant 1 (SAMR1) shows normal aging and acts as a control strain. SAMP8 and SAMR1 mice are selected by phenotype difference from one common background strain, AKR/J mice [16, 17].

SS31, a novel mitochondria-targeted antioxidant peptide, have shown the capability of reducing reactive oxygen species (ROS) and protecting mitochondria against oxidative damage [18]. Furthermore, benefi­cial effects of SS31 have been extensively studied in animal models of brain infarction, ischemia- reperfusion-induced myocardial infarction and amyotrophic lateral sclerosis (ALS) [19-21]. In our recent study, the SAMP8 mice showed cognitive decline and abnormal mitochondrial fission/fusion proteins compared to SAMR1 at 10-month-old. After the treatment of SS31 for 8 weeks, the spatial learning and memory skills were improved and the mitochondrial homeostasis was maintained [22]. However, it is unknown whether application of this compound exerted protective effects through NF-κB signaling and Nrf2/ARE pathway. Therefore, this study aims to investigate NF-κB and Nrf2 signaling underlying aging, and the effects of SS31 on age-induced damage in senescence accelerated mouse prone model.

## RESULTS

### Characterization of cytokines and intracellular reactive oxygen species (ROS) production inSAMR1, SAMP8 and SAMP8 with SS31 treatment

The amount of TNF-α, IL-1β and IL-6 protein and ROS were significantly elevated in 10-month-old SAMP8 mice compared with 10-month-old SAMR1 mice using enzyme-linked immunosorbent assay (ELISA)(Table 1). SS31treatment significantly decreased the amount of IL-1β protein and ROS in SAMP8 mice. These results suggested that inflammatory response and oxidative stress might play an important role in senescence and SS31 may target the same molecular pathway/s.

**Table 1:** Levels of cytokines (pg/mg) and ROS (/%) in SAMR1, SAMP8 and following SS31 treatment (mean ± SD)

Groups	TNF-α*	IL-1β**	IL-2	IL-6*	IL-10	ROS**
SAMR1	24.75±5.44	38.75±9.22	179.00±56.15	8.25±3.30	32.75±12.04	100.00±39.23
SAMP8	40.50±12.40^&^	57.50±3.42^&^	266.75±14.01	13.75±2.50^&^	37.25±5.68	294.83±92.91^&^
SS31	28.50±8.39	40.75±5.06^#^	233.74±74.91	10.10±3.92	37.50±12.92	208.78±56.64^#^

Overall analysis: ^*^ *P* < 0.05; ^**^ *P* < 0.01

Posthoc analysis: ^&^ *P* < 0.05: SAMP8 vs. SAMR1 mice; ^#^*P* < 0.05: SS31 vs. SAMP8 mice.

### Significantly increased NF-κB p65 mRNA expression in SAMP8 mice during aging

**Figure 1:**
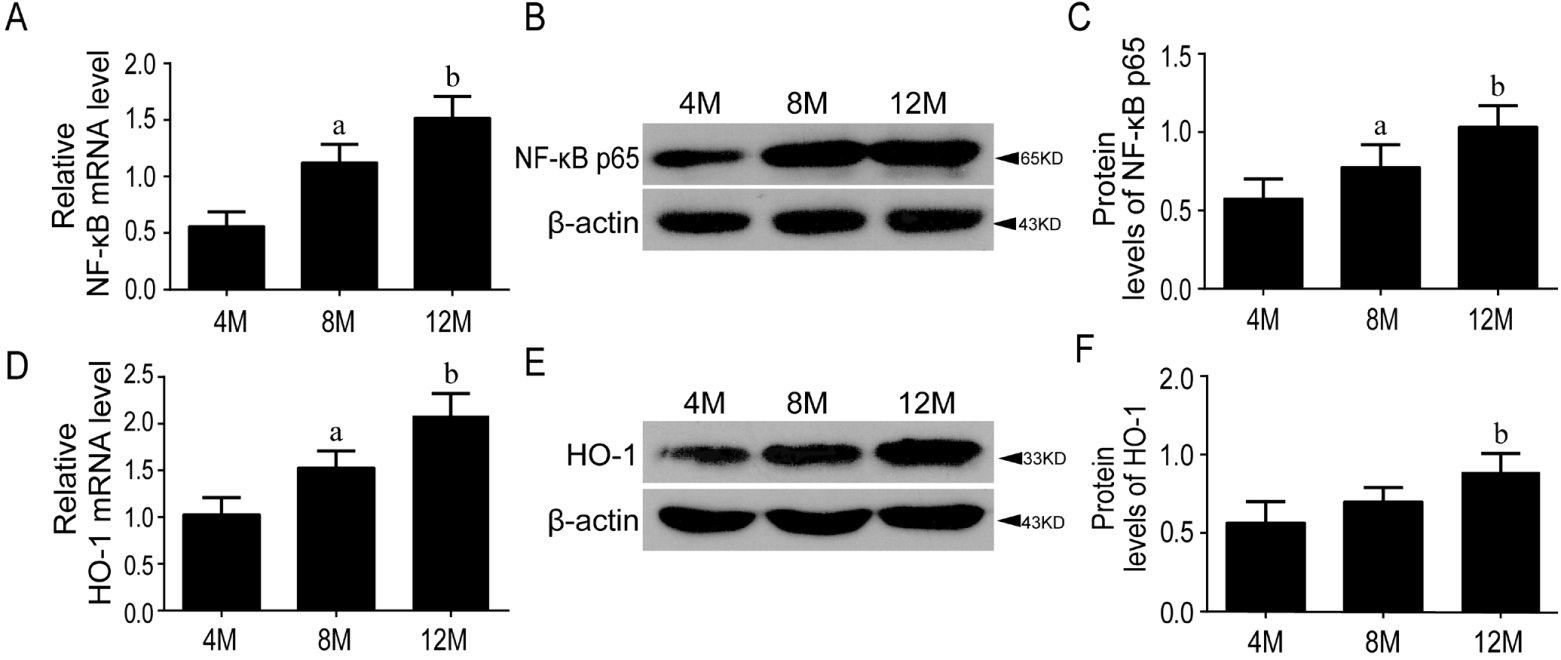
The levels of NF-κB p65 and HO-1 were increased during aging The stress-sensitive inflammatory factor NF-κB p65 increased significantly in the mRNA **A.** and protein levels of SAMP8 mice **B.**, **C.** The downstream detoxifying and antioxidant enzyme HO-1 was increased gradually both at gene expression **D.** and protein level **E.**, **F.** in aging process. 4M, 8M, 12M represent 4 month, 8 month and 12 month, respectively. Data are mean ±SD, *P < 0.05, ** *P* < 0.01.

NF-κB signaling was activated during aging process. To determine the alteration of inflammatory response in SAMP8 mice, we investigated the gene expression and protein amount of NF-κB p65 in the hippocampus of 4, 8 and 12-month-old SAMP8 mice. RT-qPCR and Western blot analyses revealed that both the gene expression and protein concentration of NF-κB p65 increased gradually with age, indicating the activation of NF-κB signaling and the increased inflammatory response in aging (Figure 1A, 1B, 1C).

#### Nrf2-ARE pathway was activated in aging

**Figure 2:**
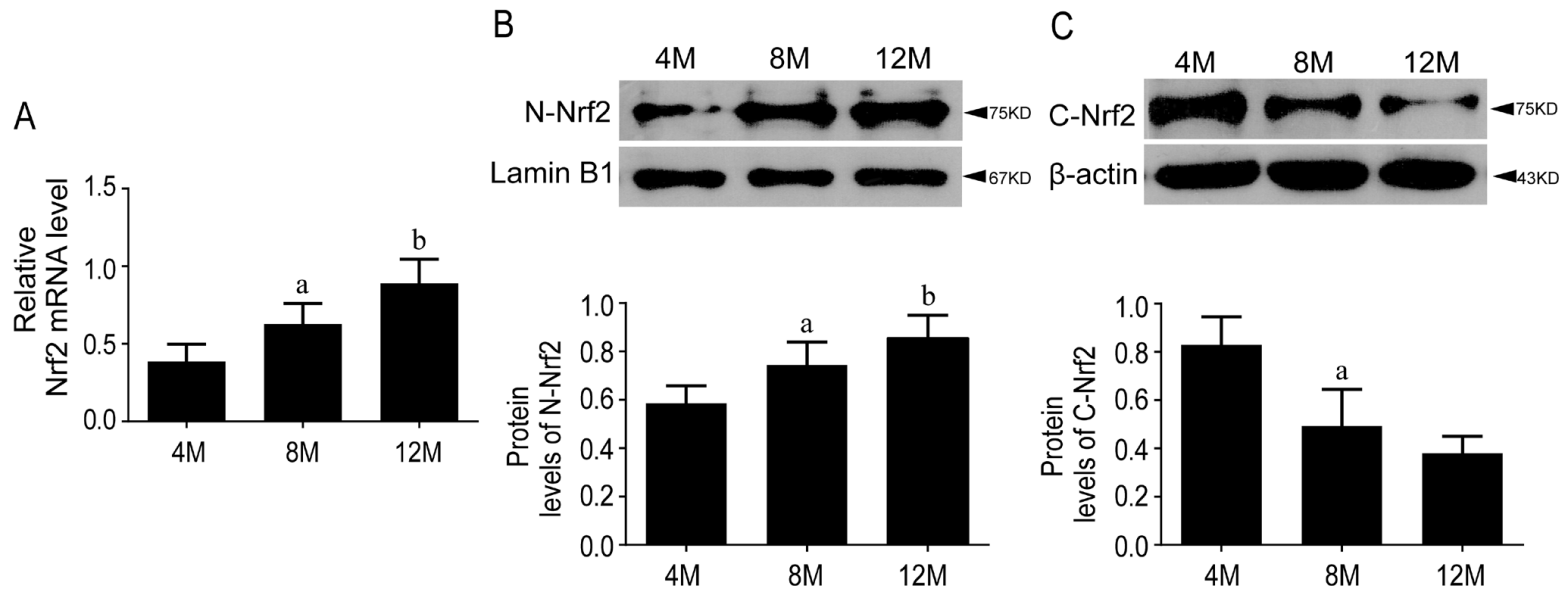
Nrf2 pathway was activated during aging in SAMP8 mice The mRNA level of Nrf2 increased gradually with age in the hippocampi of SAMP8 mice and there are significant changes among groups **A.** Protein level of N-Nrf2 were increased **B.** while C-Nrf2 were decreased **C.** with age in the hippocampi of SAMP8 mice. 4M, 8M, 12M represent 4 month, 8 month and 12 month, respectively. Data are mean ±SD, *P < 0.05, ** *P* < 0.01.

To examine the role of Nrf2 pathway in age-related mice, the gene expressions of Nrf2 and HO-1, and the protein amounts of HO-1, nuclear and cytoplasmic Nrf2 in the hippocampus of 4, 8 and 12-month-old SAMP8 were tested by RT-qPCR and Western blots. Results showed that Nrf2 and HO-1 genes were upregulated gradually with age, consistent with the significantly increased protein concentration of nuclear Nrf2 and HO-1, and the decreased cytoplasmic Nrf2, indicating that aging itself could activate Nrf2-ARE pathway in SAMP8 mice and more Nrf2 were translocated into nucleus(Figure 1D, 1E, 1F & Figure 2).

Stepwise multiple liner regression analysis showed that NF-κB p65 gene expression (R^2 ^= 0.870) was more important than HO-1 gene expression (which increased R^2^ to 0.934), demonstrating that the gene expression of NF-κB was an important factor in aging process (Table 2).

**Table 2:** Model summary of stepwise multiple liner regression analysis

model	parameters	R	R^2^	adjusted R^2^
Aging group	r. NF-κB	0.933	0.870^a^	0.862
	r.HO-1	0.967	0.934^b^	0.926
				
SAMR1VS. SAMP8	r.NF-κB	0.889	0.790^c^	0.769
	p.C-Nrf2	0.968	0.937^d^	0.923
	r.HO-1	0.992	0.983^e^	0.977
				
SS31 group	r.NF-κB	0.901	0.813^f^	0.794
	r.Nrf2	0.990	0.981^g^	0.976

r.: mRNA level; p.: protein level.

a: Predictors: (Constant): r.NF-κB;

b: Predictors: (Constant): r.NF-κB, r.HO-1;

c: Predictors: (Constant): r.NF-κB;

d: Predictors: (Constant): r.NF-κB, p.C-Nrf2;

e: Predictors: (Constant): r.NF-κB, p.C-Nrf2, r.HO-1;

f: Predictors: (Constant): r.NF-κB;

g: Predictors: (Constant): r.NF-κB, r.Nrf2.

### Significant increase of NF-κB p65 in SAMP8 relative to SAMR1 at the age of 10-month-old

#### NF-κB signaling was activated in SAMP8 at 10-month-old

**Figure 3:**
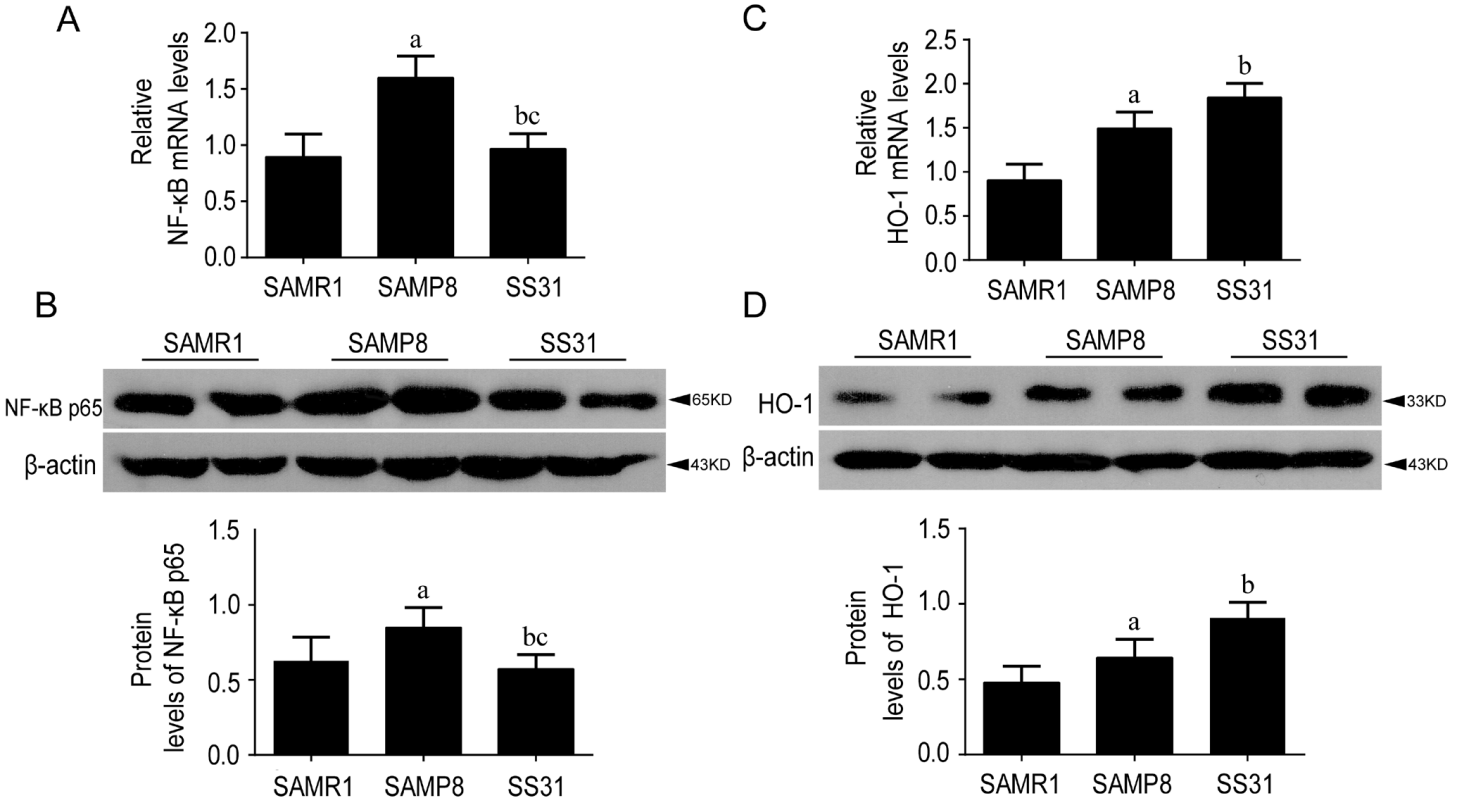
The levels of NF-κB p65 and HO-1 were different between SAMR1 and SAMP8, While SS31 exerted anti-inflammatory effects Compared with SAMR1, the gene expression **A.** and protein levels **B.** of NF-κB p65 increased significantly in the SAMP8 compared to SAMR1 at the age of 10 months old. After SS31 administration, they were decreased and recovered to SAMR1 level (A, B). The downstream detoxifying and antioxidant enzyme HO-1 were increased both at gene expression **C.** and protein levels **D.** relative to SAMR1. After SS31 administration, these parameters were increased further. Data are mean ±SD, *P < 0.05, ** *P* < 0.01.

Gene expression and protein amount of NF-κB p65 in the hippocampus of 10-month-old mice were tested to detect the differences in NF-κB signaling pathway between SAMP8 and SAMR1. Results showed that both the gene expression and protein amount of NF-κB p65were increased significantly in SAMP8 compared to SAMR1, indicating the activation of NF-κB signaling in senescence accelerated mice(Figure 3A, 3B).

#### Nrf2-ARE pathway was activated in SAMP8 at 10-month-old

**Figure 4:**
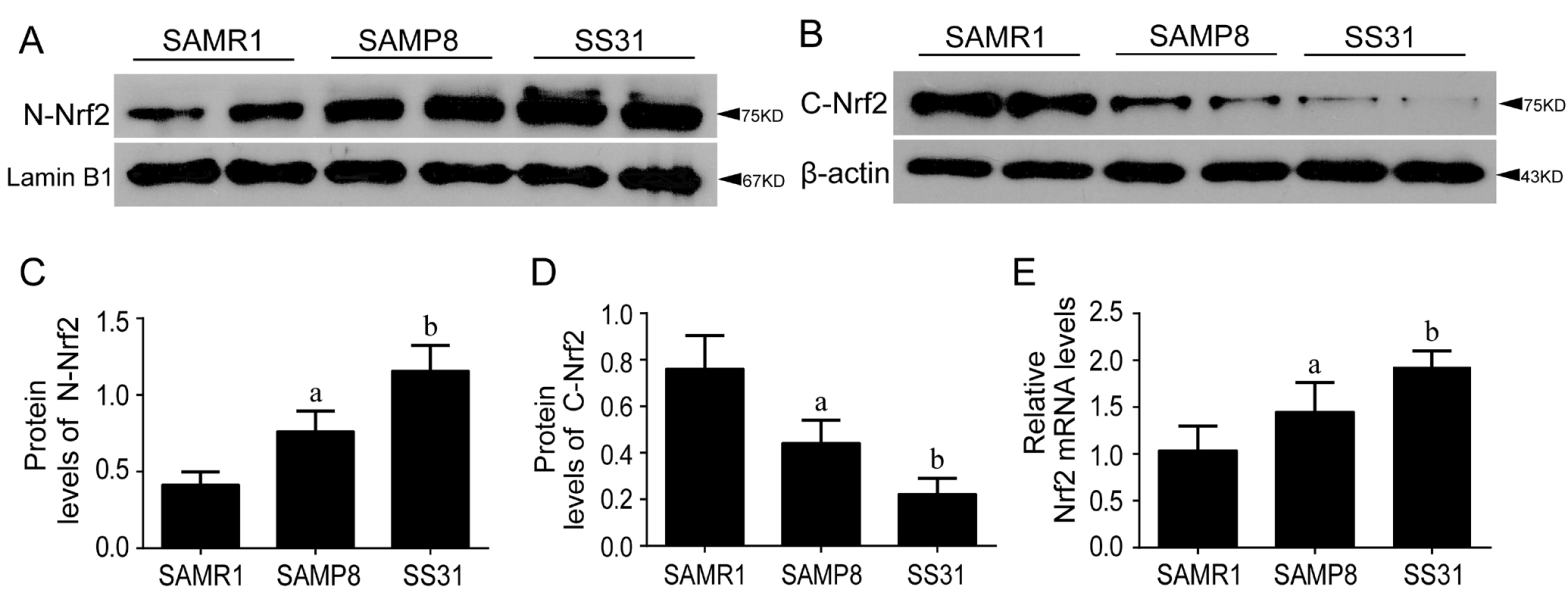
Nrf2-ARE pathway was activated in SAMP8 relative to SAMR1 and exerted anti-oxidant effects after administration of SS31 Compared with SAMR1, the protein levels of N-Nrf2 **A.**, **C.** and gene expression levels of Nrf2 **E.** were increased in the hippocampi of SAMP8 mice at the age of 10 months old, accompanied by decreased protein levels of C-Nrf2 **B.**, **D.** After SS31 administration, both mRNA of Nrf2 (E) and protein levels of N-Nrf2 (A, C) were increased further, with the further decreased C-Nrf2 (B, D). Data are mean ±SD, *P < 0.05, ** *P* < 0.01.

Nrf2 and HO-1 genes expression and the protein amount of HO-1, nuclear and cytoplasmic Nrf2 content were examined by RT-qPCR and Western blot in the hippocampus of 10-month-old SAMP8 and SAMR1. Results showed that both gene expression and protein concentration of nuclear Nrf2 were increased in SAMP8 compared to SAMR1, accompanied by decreased protein concentration of cytoplasmic Nrf2 (Figure 4). The downstream detoxifying and antioxidant enzyme HO-1 appeared the same pattern as Nrf2 (Figure 3C, 3D).

According to stepwise liner regression analysis, NF-κB p65 gene expression (R^2 ^= 0.790) was more important predictive parameter than others, such as cytoplasmic Nrf2 protein amount(which increased R^2^ to 0.937), or HO-1 gene expression (which further increased R^2^ to 0.983), for the aged model of SAMP8 compared to its counterpart SAMR1(Table 2).

### Significant decrease NF-κB p65 gene expression in SAMP8 mice following SS31 treatment

#### NF-κB signaling was suppressed by SS31 in SAMP8 mice

The alteration of NF-κB p65 was tested by RT-qPCR and Western blot after the treatment of SS31 in SAMP8.

Compared with saline-treated SAMP8, the gene expression and protein concentration of NF-κB p65 were decreased significantly after SS31 treatment (*p*<0.05, Figure 3A). No significant differences were observed between SAMR1 and SS31 group both in the mRNA (Figure 3A) and protein amount (Figure 3B) of NF-κB p65. Our data showed that SS31 could suppress NF-κB p65 signaling and exerted anti-inflammatory role in SAMP8 mice.

#### SS31activated Nrf2-ARE pathway in SAMP8 mice

We examined whether the intervention ofSS31 influenced the activation of Nrf2 pathway. Compared with saline-treated SAMP8, SS31 significantly increased the gene expression of Nrf2 and HO-1, accompanied by an increase in nuclear Nrf2, HO-1 protein and a decrease in cytoplasmic Nrf2 protein (Figure 3C, 3D, and Figure 4). These results suggested that the administration of SS31 promoted Nrf2 activation and developed anti-oxidant role in SAMP8 mice.

According to stepwise multiple liner regression analysis, NF-κB p65 gene expression (R^2 ^= 0.813) was more important than Nrf2 gene expression (which increased R^2^ to 0.981) after the treatment of SS31 (Table 2).

## DISCUSSION

Pathological aging and age-related disorders have become major health concerns due to the increase in life expectancy [23]. Therefore, understanding the mechanisms that are responsible for age-related neurodegeneration may provide new perspectives on the etiology of such dysfunctions and promote therapeutic strategies for brain damage. The aim of the current study was to determine NF-κB signaling and Nrf2/ARE pathway related to inflammation and oxidation on the premature neurologic dysfunction in senescence accelerated mice. SAMP8 mouse model exhibits cognitive and emotional disturbances at a young age, probably due to pathological mechanisms such as inflammatory and oxidative signaling pathways [24]. Furthermore, this murine strain, SAMP8, has been described as a nature, non-transgenic tool for accelerated brain senescence [25].

In this study, we found significant increases of both gene expression and protein concentration of NF-κB p65 in 10-month-old SAMP8 mice relative to SAMR1 (Figure 3A, 3B) and they also increased during the process of aging in SAMP8 mice (Figure 1A, 1B, 1C). It has been widely accepted that inflammation plays a critical role in aging and neurodegeneration, in which the major signaling proteins include cytokines, growth factors, as well as chemokines [3]. In this study, we showed the levels of pro-inflammatory cytokines IL-1β, TNF-α, IL-6 and other inflammatory mediators in the brain of SAMP8 were increased at 10 months of age compared with those at SAMR1, which is consistent with previous study [26]. The expression of these pro-inflammatory and pro-apoptotic genes can be initiated by NF-κB in response to oxidative stress, evidenced by elevated ROS in SAMP8 (Table 1). NF-κB might be constitutively activated by a shift in intracellular redox state during aging, invoking long-lastinginflammatory response contributing to many age-related chronic diseases [10]. Our results showed that the gene expression of NF-κB was the most strongly associated with age, which shared the same conclusion with a review which tested gene expressions from nine different types of tissues in mice and humans [27]. These data indicated NF-κB as a promising target for slowing down the aging process.

Our data showed that both gene expression and protein amount of Nrf2 were higher in SAMP8 compared with SAMR1 at 10-month-old (Figure 4A, 4C, 4E). The gene expression of Nrf2 and nuclear component of Nrf2 protein were increased gradually with age in SAMP8 (Figure 2A, 2B). The expression of HO-1 appeared the same pattern as Nrf2 (Figure 1D, 1E, 1F & Figure 3C, 3D), suggesting the activation of Nrf2/ARE pathway during aging in SAMP8 mice. In contrast, some studies showed that the levels of HO-1 in SAMP8 are lower than in SAMR1 [28, 29], possibly due to the SAMP8 mice used with different age and gender. HO-1, modulated by Nrf2/ARE pathway, is a rate-limiting enzyme in the oxidative degradation of heme to biliverdin, free iron and carbon monoxide [30]. Accelerated aging elicits cytoprotective and antioxidant response in hippocampus by activating Nrf2/ARE pathway gradually. Nrf2 is a principal mediator for primary cellular defense against cytotoxic effects for the central nervous system (CNS), including pathways for DNA repair, molecular chaperones, antioxidants, anti-inflammatory response, and proteasome systems [22]. The Nrf2 dependent self-protective antioxidant response is a complex and highly orchestrated pathophysiological process. As shown in this study (Table 1), ROS is about three fold increased in SAMP8.Upon oxidative stress, mammalian cells will immediately initiate a highly efficient cytoprotective machinery to protect cells against the harmful effects of ROS, which might reflect the increased Nrf2 levels in SAMP8 mice in this study, although other studies showed the opposite effects [31, 32], which might indicate gradual activation of Nrf2, a general mechanism for self-protection in multiple organ systems upon chronic injury. Much of this machinery stems from Nrf2 antioxidant pathway, which is the primary cellular defense signaling [14, 33, 34].

The expression modulation of two transcription factors, NF-κB and Nrf2, which regulates proinflammation (such as IL-1β, TNF-α) and anti-oxidant response (such as HO-1, NQO1), respectively. NF-κB and Nrf2 pathways share common effectors and regulatory points and NF-κB pathway can be inhibited by several Nrf2 activators [35]. A large number of pathological stimuli, such as cigarette smoke, lipopolysaccharide (LPS), reactive oxygen species (ROS) and oxidized low-density lipoprotein, activates both NF-κB signaling and Nrf2-ARE pathway [7, 36-38].

Following the treatment of SS31, the NF-κB induction was recovered to the similar amount of wildtype SAMR1(Figure 3A, 3B). In addition, more Nrf2 genes were expressed and more cytoplasmic Nrf2 were translocated into nucleus to guard against oxidative stress (Figure 4), accompanied by increased concentrations of HO-1 (Figure 3C, 3D). This demonstrated the suppression of NF-κB signaling and activation of Nrf2-ARE pathway by SS31, therefore exerting anti-inflammatory and anti-oxidant roles. SS31 can penetrate into the inner mitochondrial membrane selectively, where it scavenge ROS generated by the electron transport chain and inhibit oxidation of low-density lipoprotein, thereby protecting mitochondria against Ca2+-induced mitochondrial permeability transition, swelling and release of cytochrome C and prevent t-butylhydroperoxide-induced apoptosis [19]. Additional evidence includes SS31 applied in Tg2576 mice to protect N2a cells and primary neurons against Aβ-induced neurotoxicity [39]. Mitochondrial abnormalities induced by Aβ are early events and play critical roles in AD pathogenesis [40]. Furthermore, SS31 is found to be protective against Aβ-induced abnormal mitochondrial dynamics [41].

Our results suggest that accelerated aging could activate both NF-κB and Nrf2-ARE signaling pathways, and SS31 could exert anti-inflammatory and anti-oxidant roles mainly by suppressing NF-κB signaling and activating Nrf2-ARE pathway. Although multiple molecules involved in oxidative stress and inflammation are changed during aging process, RELA (NF-κB p65)gene expression was an important independent variable among groups by stepwise liner regression analysis (Table 1). The inflammatory factor, mRNA expression of NF-κB p65, was more important during aging process and SS31 exerted protective effects mainly by reducing the gene expression of NF-κB p65. In summary, the present data suggest that aging is associated with significant alterations in the genes expression involved in inflammation and oxidative stress in the brain. SS31 administration might be able to rescue SAMP8 mice from the accelerated aging process.

## MATERIALS AND METHODS

### Ethics approval

The handling of mice and experimental procedures were approved by the guidelines of the National Institutes of Health Guide for the Care and Use of Laboratory Animals.

### Animals

Thirty male SAMP8 and 6 male SAMR1 mice were used in this study. All mice, purchased from the Tianjin University of Traditional Chinese Medicine (Production license number: SCXK (Tianjin) 2008-0006, Tianjin, China), were maintained in a 12:12 (light-dark) cycle at a constant temperature (22±2°C), with free access to standard diet and water. The experiments were carried out by two study groups in the Clinical Research Center of Hebei General Hospital.

### Study design

The average longevity of SAMP8 is 10-17.2 months. The cognitive function of SAMP8 didn’t change until 4 months, declines in 8 months and impaired severely in 12 months, which indicates that 4, 8, and 12 months old SAMP8 are proper time for detecting gradually impaired learning and memory abilities. In addition, 8-month-old SAMP8 mice showed mild cognitive decline, which is suitable for pharmacological intervention [42,43]. On this accountant, 4, 8, and 12 months old SAMP8 mice were selected in the first part of our study to detect the NF-κB and Nrf2 signaling underlying accelerated aging. At the second set of experiment, we chose 8-month-old SAMP8 mice (*n* = 6) with an administration of SS31 (1nM, 5 mg/kg/day as optimal [21] by intraperitoneal injection for 8 weeks) to investigate possible therapeutic mechanisms for senescence.

Part 1: 18 male SAMP8 mice were randomly separated into three groups (*n* = 6) and cultivated till 4, 8 and 12 months old respectively.

Part 2: 8-month-old male SAMR1 (*n* = 6) and SAMP8 (*n* = 12) were cultivated, and 6 SAMP8 mice were randomly chosen to get an intraperitoneal injection with SS31. The small molecular peptide SS31 (D-Arg-Dmt-Lys-Phe-NH2; 2′,6′- dimethylty-rosine) was synthesized as described previously and was diluted with saline before use, at a concentration of 1nM. The other groups were treated with normal saline. All treatments with SS31 were used at a dose of 5 mg/kg/day by intraperitoneal injection. After 8 weeks, there were 3 groups of 10-month-old mice: SAMR1 saline-treated group (*n* = 6, SAMR1), SAMP8 saline-treated group (*n* = 6, SAMP8) and SAMP8 SS31-treated group (*n* = 6, SS31).

Animals were sacrificed with 1% pentobarbital sodium at a dose of 50mg/kg for tissue harvesting. The hippocampus were dissected from brains, frozen immediately in liquid nitrogen and stored at -80°C, which were for RNA and protein extraction. The gene expressions of NF-κB, Nrf2 and HO-1 were tested using RT-qPCR. The total protein amounts of NF-κB and HO-1, as well as the protein concentrations of nuclear and cytoplasmic Nrf2 were tested using western blots.

### ELISA analysis for cytokines

Protein concentration of TNF-α, IL-1β, IL-2, IL-6, and IL-10 was measured in tissue lysates from hippocampus using an ELISA kit (ExCell Biotech). Following the instruction, 100 μl (1μg/μl) tissue lysates were added to appropriate wells and incubated overnight at 4°C with gentle shaking. On the next day, the solution was discarded and the plates were washed four times with 1× wash solution (ExCell Biotech). Plates were incubated for 1 h at room temperature with detection antibody and washed four times. Then, 100 μl HRP streptavidin was added to each well and incubated for 45 min at room temperature. Following four times washes, 100μl TMB (ExCell Biotech) was added to each well and incubated for 30 min at room temperature in the dark. Finally, 50 μl stop solution (ExCell Biotech) was added, and the absorbance was measured at 450 nm immediately.

### Intracellular ROS measurements

About 50 μg brain tissues from temporal lobe were dissected and cut into small pieces (1-2 mm^3^). 0.25% trypsin was added and incubated at 37 °C with gentle shaking for 20 minutes for digestion. The single cell suspension was collected by pouring the solution through a double 100 mesh stainless steel cell filter. The collection was then centrifuged at 1000 g for 10 min. The supernatant was discarded and cell pellets were collected, washed with phosphate buffer for 2 to 3 times and re-suspended. The cells solution was then incubated with 10 μM DCFH-DA at 37°C in CO_2_ incubator for 60 min. The fluorescence was measured in a flow cytometry (Spectra Max GEMINI XPS, Molecular Devices, USA) with excitation at 488 nm and emission at 525 nm. The results were expressed as percentages relative to SMAR1 mice.

### Western blotting

Nuclear and cytosolic fractions were extracted from the hippocampus using Nuclear-Cytosol Extraction Kit (TDY, Biotech CO., Ltd, Beijing), and total proteins were isolated by Radio Immune Precipitation Assay (RIPA) buffer (Solarbio, Beijing, China) following the manufacture's protocols. The protein concentration was measured by the BCA protein assay (Pierce, Rockford, USA). Equal amounts of protein extraction (25-50μg) were loaded onto 10%-15% SDS/PAGE gels and transferred onto 0.45 μm PVDF membranes (Millipore Corporation, Billerica, USA), which were needed to be first blocked with 5% skimmed milk for 2hrs at room temperature, then incubated overnight (4°C) with primary antibodies: NF-κB p65 (1:800, Santa Cruz Biotechnology, USA), Nrf2 (1:500, Santa Cruz Biotechnology, USA), HO-1 (1:500, Protein Tech Group, China), β-actin (1:5000, Protein Tech Group, China, internal control), and Lamin B1 (1:1000, Protein Tech Group, China, intra-nuclear control). After washing with TBST (50 mm Tris, 150 mm NaCl, 0.1% Tween-20, PH 7.4) the next day, the membranes should be incubated with secondary antibody (1:5000, goat-anti-rabbit-HRP, Protein Tech Group, China) for 2hrs at room temperature. Immunoreactive bands were visualized by X-ray film exposure with ECL kit (Shanghai Sangon, China). The signal intensities of the entire bands for different proteins were analyzed by Image J (version 1.30v, Wayne Rasband, National Institutes of Health, Bethesda, MD).

### Real-time reverse transcription-quantitative PCR (RT-qPCR)

Total RNA of the hippocampus was extracted using Trizol reagent (Tiangen, China). Aliquots of total RNA were reverse-transcribed into cDNA (*n* = 6 in each group) using the Transcriptor First Strand cDNA synthesis Kit (Transgene, China) according to manufacturer's recommendation. RNA was dissolved in RNase-free water. Primers were synthesized by Shanghai Sangon Biological Engineering Technology Company Limited. The gene order was proven to be correct in GenBank. The RT-qPCR conditions included: 1 cycle of initial denaturation (95°C for 10 min), 40 cycles of amplification (95°C for 10 s and 60°C for 20 s), and a cooling period (50°C for 5 s). The final data are relative mRNA concentrations normalized to GADPH. Experiments were repeated for 3 times. The primer sets are as follows: NF-κBp65 (forward primer: 5′-GCG AGA GAA GCA CAG ATA CCA-3′, reverse primer: 5′-GGT CAG CCT CAT AGT AGC CAT-3′), Nrf2 (forward primer: 5′-AGT GAC TCG GAA ATG GAG GAG -3′; reverse primer:5′-TGT GCT GGC TGT GCT TTA GG -3′), HO-1 (forward primer: 5′-GCT GGT GAT GGC TTC CTT GT-3′; reverse primer: 5′-ACT GGG TTC TGC TTG TTG CG-3′), GAPDH (forward primer: 5′-TGA ACG GGA AGC TCA CTG G-3′; reverse primer: 5′-GCT TCA CCA CCT TCT TGA TGT C-3′(internal control).

### Statistics analysis

Statistics analyses were performed using SPSS for windows version 21.0 (International Business Corporation Inc., Amonk, NY) and GraphPad Prism Software for Science, Version 5.0c (San Diego, CA). One-way ANOVA with posthoc analysis was applied to compare the amounts of molecular targets among and between groups. Stepwise multiple linear regression analysis was carried out to determine the parameters which were independently correlated to the variations. Variables entered into the model were important parameters among all molecules tested. With *p* value<0.05, the difference was considered statistically significant.
